# Structures of the peptidase-containing ABC transporter PCAT1 under equilibrium and nonequilibrium conditions

**DOI:** 10.1073/pnas.2120534119

**Published:** 2022-01-24

**Authors:** Virapat Kieuvongngam, Jue Chen

**Affiliations:** ^a^Laboratory of Membrane Biology and Biophysics, The Rockefeller University, New York, NY 10065;; ^b^HHMI, Chevy Chase, MD 20815

**Keywords:** ABC transporters, lowest energy state, rate-limiting step

## Abstract

ATP-binding cassette (ABC) transporters are ubiquitous molecular pumps that transport a broad range of substrates across biological membranes. Although the structure and function of ABC transporters has been studied extensively, our understanding of their energetics and dynamics remains limited. Here, we present studies of the peptidase-containing ABC transporter 1 (PCAT1), a polypeptide processing and secretion ABC transporter that functions via the classic alternating access mechanism. PCAT1 is a homodimer containing two peptidase (PEP) domains, two transmembrane domains, and two nucleotide-binding domains (NBDs). Using cryo-electron microscopy, we analyzed the structures of wild-type PCAT1 under conditions that either prevent or permit ATP hydrolysis and observed two completely different conformational distributions. In the presence of ATP but absence of Mg^2+^, PCAT1 adopts an NBD-dimerized, outward-facing conformation. The two PEP domains are dissociated from the transporter core, preventing uncoupled substrate cleavage. The addition of Mg^2+^ to promote ATP hydrolysis shifts the majority of the particles into NBD-separated, inward-facing conformations. Under this ATP turnover condition, only a small fraction of PCAT1 adopts the NBD-dimerized conformation. These data give rise to two mechanistic conclusions: 1) the ATP-bound, NBD-dimerized conformation is the lowest energy state, and 2) the rate-limiting step in the PCAT1 transport cycle is the formation of the NBD dimer. The thermodynamic conclusion is likely a general property shared by many ABC transporters. The kinetic bottleneck, however, varies from transporter to transporter.

## Significance

Recent advances in cryo-electron microscopy (cryo-EM) enabled us to determine multiple structures from the same sample. At equilibrium, the Boltzmann distribution law can be applied to identify the lowest energy state of the protein. We found that the conformational distributions of PCAT1, the peptidase-containing ATP-binding cassette (ABC) transporter 1, are completely different in the presence and absence of the Mg^2+^ ion. This difference reflects energy inflow from ATP hydrolysis, shifting the system out of equilibrium. The conformational distribution under ATP turnover condition is determined by the transition rates along the transport pathway rather than the energy of each state. This study demonstrates how cryo-EM data can be used to understand thermodynamic and kinetic properties of an active transporter.

ATP-binding cassette (ABC) transporters are molecular machines that convert the chemical energy stored in ATP to the electrochemical potential of a substrate. Peptidase-containing ABC transporters (PCATs) in gram-positive bacteria function both as maturation proteases and exporters for biofilm formation, quorum sensing, or antimicrobial polypeptides (1, 2). Some PCATs are also found in gram-negative bacteria, interacting with a periplasmic adaptor protein and an outer membrane porin to secrete antimicrobial peptides called microcins (3). Substrates of PCATs are synthesized as precursors with an N-terminal leader peptide. Proteolytic cleavage of the leader peptide at the conserved double-glycine motif is necessary for secretion of the cargo peptide (4).

Previously, the structure of PCAT1 from *Clostridium thermocellum* was determined in three conformational states: an apo conformation in the absence of substrate and nucleotides (5), a substrate-bound nucleotide-free conformation trapped with a proteolytic-deficient mutant C21A (6), and an ATPγS-bound conformation trapped with a hydrolytic-deficient mutant E648Q (5). In the absence of ATP and substrate, PCAT1 exhibits an inward-facing (IF) conformation. The two transmembrane domains (TMDs) form a large α-helical barrel sufficient to accommodate the entire cargo peptide. The nucleotide-binding domains (NBDs) are separated, and the peptidase (PEP) domains dock onto the intracellular openings of the translocation pathway. In the presence of the substrate CtA but absence of ATP, PCAT1 also forms an IF conformation (6). Two substrates are bound via their leader peptides, but only one of them is positioned for cleavage and translocation (6). Finally, a low-resolution crystal structure of the hydrolysis-incompetent mutant E648Q in complex with ATPγS shows a closed NBD dimer and an occluded translocation pathway (5). The two PEP domains were not resolved in the structure, suggesting that they are flexibly attached to the transporter core (5).

A limitation of the aforementioned studies is that the structures were obtained through biochemical “trapping”, providing only snapshots during the transport cycle. To reduce conformational heterogeneity, the transport cycle was stalled by either omitting ATP or mutating the catalytic residues. With recent advances in cryo-electron microscopy (cryo-EM), it is now possible to analyze a transporter under active turnover conditions to understand its conformational distribution (7–10). In this work, we determined the structures of wild-type PCAT1 (*wt*PCAT1) in the presence of substrate and ATP with or without Mg^2+^. These analyses revealed a major change of the conformational distribution upon ATP hydrolysis, reflecting the thermodynamic and the kinetic properties of the transport cycle.

## Results

### Structure Determination of *wt*PCAT1 in the ATP-Bound, Prehydrolytic Conformation.

**Fig. 1.**
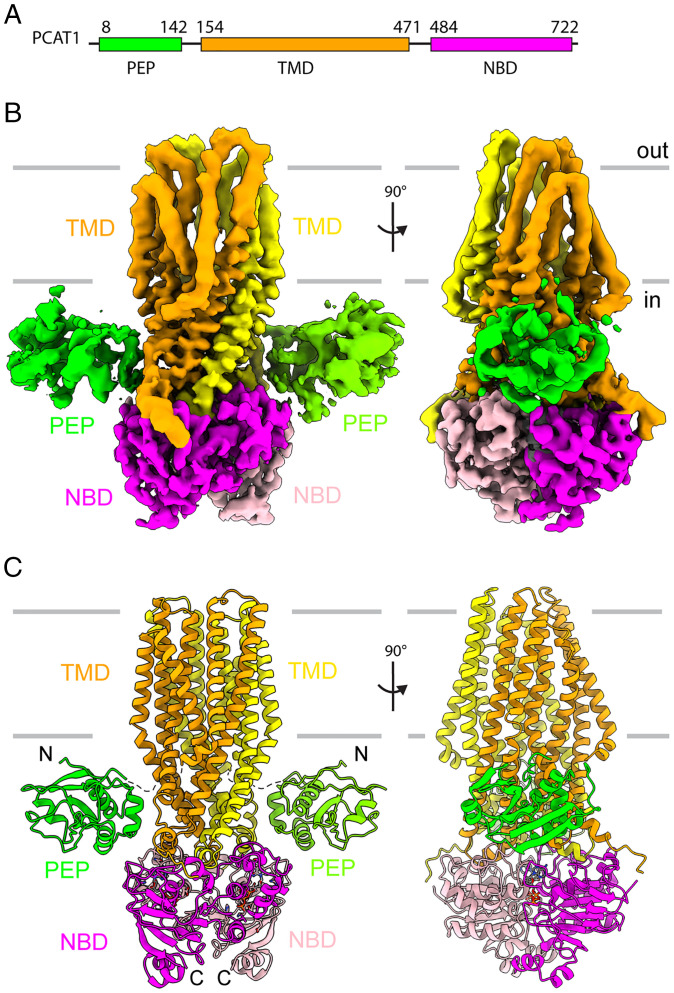
The structure of PCAT1 in equilibrium with ATP. (*A*) Cartoon illustration depicting the domain organization of PCAT1. (*B*) Two orthogonal views of the cryo-EM density map. The density is color coded and labeled by domains: magenta, NBD; orange, TMD; and green, PEP. (*C*) The ribbon representations of the overall structure.

Previous studies showed that addition of ATP in the absence of Mg^2+^ inhibits substrate cleavage of PCAT1 (5). To obtain a structural basis for this biochemical property, *wt*PCAT1 was vitrified in the presence of excess substrate and ATP. Mg^2+^ ion was omitted to prevent ATP hydrolysis. Cryo-EM reconstruction resulted in a predominant conformation at an overall resolution of 4.5 Å (Fig. 1 and *SI Appendix*, Fig. S1 and Table S1). Density modification further improved the map, showing side chain densities for the dimerized NBDs as well as ligand densities that are consistent with ATP (Fig. 1*B* and *SI Appendix*, Fig. S1*F*). The TMDs have well-defined densities in the intracellular region. The outer leaflet region exhibits a clear secondary feature with little side chain information, indicating higher degrees of flexibility (Fig. 1). Densities corresponding to the two PEP domains are visible as two amorphous blobs connected to the transporter core (Fig. 1*B*). No density corresponding to the substrate is observed, possibly due to the limited resolution of the PEP domains.

**Fig. 2.**
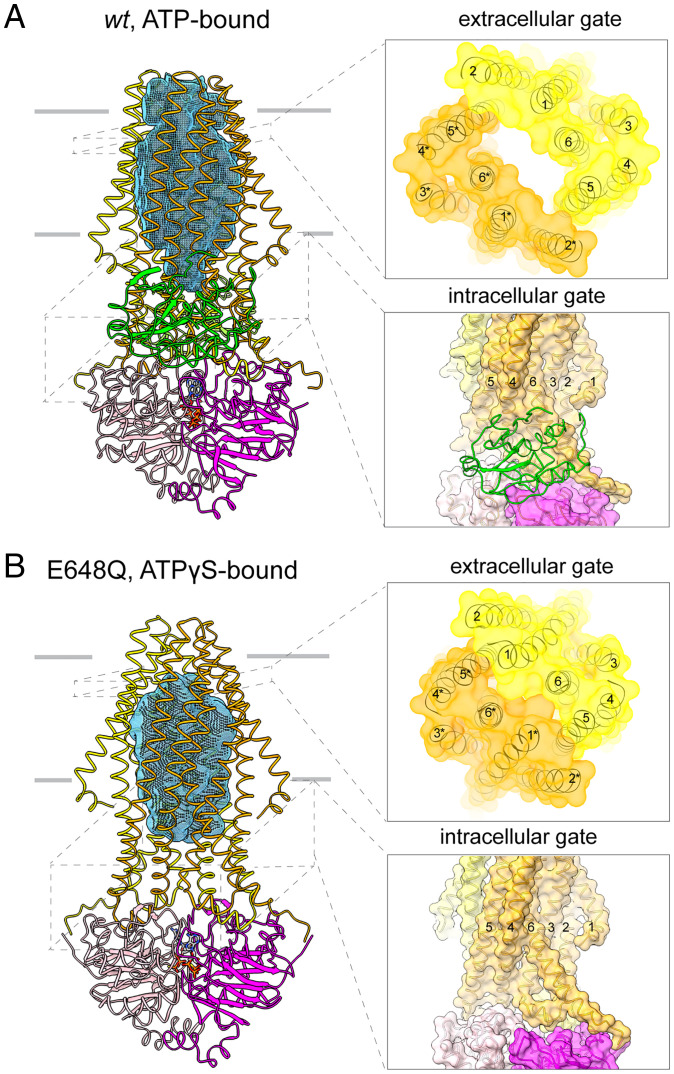
Structural comparison of the ATP-bound *wt*PCAT1 (*A*) and the ATPγS-bound E648Q mutant (*B*). (*Left*) The overall structure. The TM cavity, generated using a probe of 3-Å radius, is shown as a blue mesh. (*Right*) Zoomed-in views of the extracellular gate (viewed from extracellular space) and the intracellular gate (viewed as the overall structure). TM helices forming the gates are labeled.

In the presence of ATP without Mg^2+^, *wt*PCAT1 adopts an outward-facing (OF) conformation in which the NBDs form a closed dimer, and the translocation pathway opens to the extracellular space (Figs. 1 and 2*A*). The intracellular, lateral opening of the translocation pathway observed in the IF conformations is completely closed (Fig. 2*A*). The PEP domains are flexibly attached to the core transporter, with no defined interactions with the TMDs or the NBDs.

Structural comparison with the ATPγS-bound E648Q mutant (5) revealed two major differences (Fig. 2). First, in the crystal structure of the E648Q mutant, the PEP domains are entirely invisible, while the present cryo-EM reconstruction displays clear densities for the PEP domains, albeit at a low resolution. Second, the E648Q structure exhibits an occluded conformation in which the transmembrane (TM) cavity is closed to both sides of the membrane (Fig. 2*B*). These differences may be attributed to energetic differences introduced by the E648Q mutation, the absence of Mg^2+^ or presence of substrate in the cryo-EM sample, or the influence of crystal packing.

### Structure Determination of PCAT1 under Active Turnover Conditions.

**Fig. 3.**
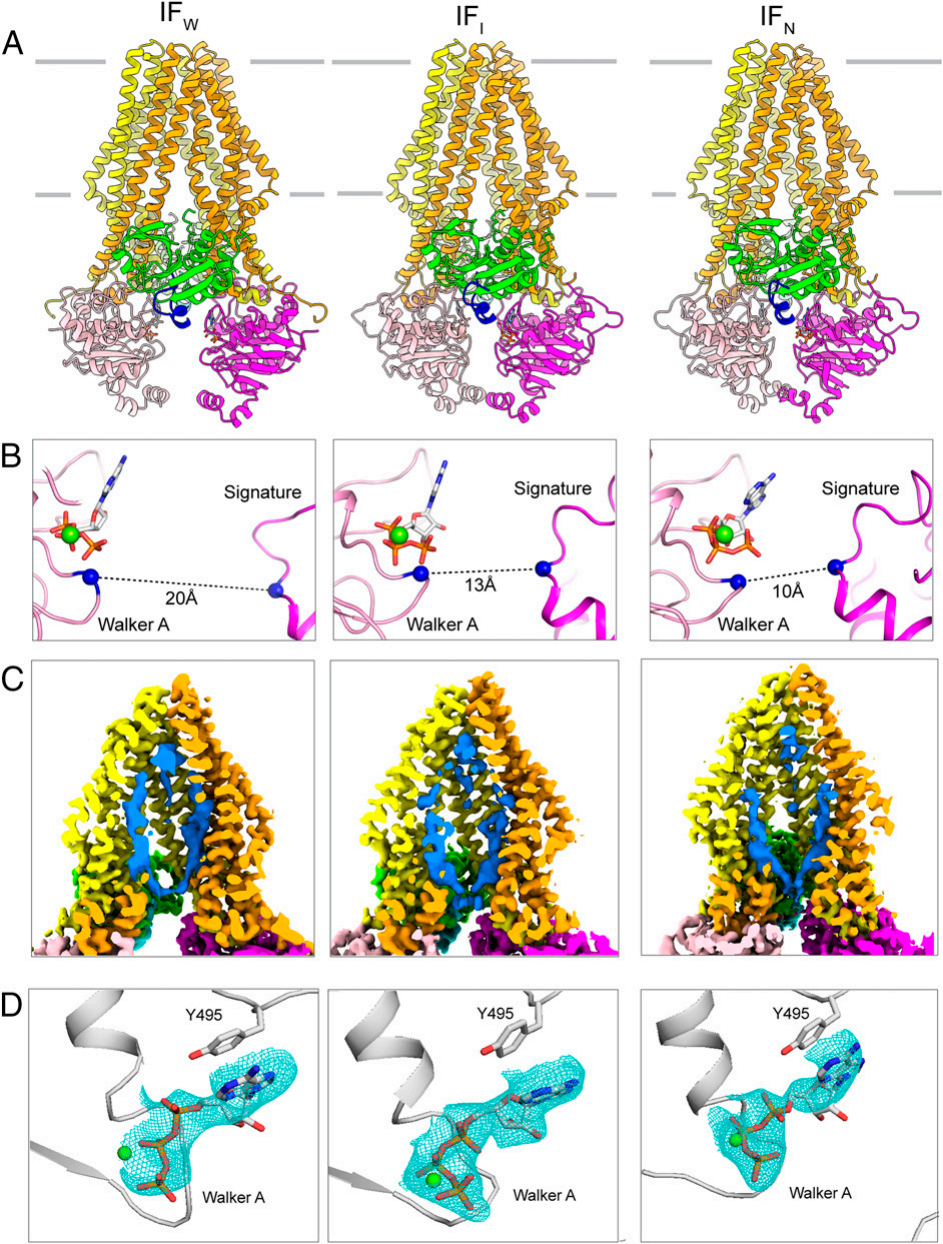
Three IF conformations determined under ATP turnover conditions. (*A*) The overall structure in ribbon representations, color coded by domains: blue, CtA; magenta, NBD; orange, TMD; and green, PEP. (*B*) Zoom-in views showing that the NBDs are separated to different degrees. The Cα distances between G522 in the Walker A motif of one NBD and S624 in the ABC signature motif of the other NBD are indicated. (*C*) Cross-sectional views of the cryo-EM density maps. The cargo density is shown in blue. (*D*) Zoom-in views of the density corresponding to ATP-Mg^2+^, shown as a blue mesh.

To analyze the conformational states of PCAT1 under ATP turnover conditions, cryo-EM samples were prepared using *wt*PCAT1 mixed with twofold molar excess of the substrate CtA, 10 mM ATP-Mg^2+^, and the creatine phosphate ATP-regenerating system. In marked contrast to the sample prepared in the absence of Mg^2+^, multiple distinct conformations were observed (Fig. 3 and *SI Appendix*, Figs. S2 and S3 and Table S2). Among the 671,000 particles analyzed by three-dimensional (3D) classification, ∼83% exhibit IF conformations with different degrees of NBD separation, 14% are in an NBD-dimerized configuration, and the remaining 3% do not show any protein features (*SI Appendix*, Fig. S2). Subsequent refinement resulted in a low-resolution (6.6-Å) NBD-dimerized structure and three molecular structures of NBD-separated IF conformations, referred to as IF wide (IF_W_, 4.1 Å), intermediate (IF_I_, 3.7 Å), and narrow (IF_N_, 3.7 Å), respectively (Fig. 3 and *SI Appendix*, Fig. S2).

The three IF structures share a similar overall conformation, in which the two PEP domains dock symmetrically onto the intracellular openings of the TM cavity (Fig. 3). The leader peptide (residues 8 to 24) of the substrate forms an L-shaped structure wrapping around the PEP domain (*SI Appendix*, Fig. S4*A*). The double-glycine cleavage site (G23/G24) is positioned on a narrow cleft on PEP in close proximity to the catalytic triad residues C21, H99, and D115 (*SI Appendix*, Fig. S4*B*).

Inside the TM cavity, amorphous densities were observed in all three reconstructions (Fig. 3*C*). Studies of the C21A mutant have shown that a similar density corresponds to a cargo peptide inserted into the translocation pathway (6). In contrast to the C21A mutant where the catalytic cysteine is absent, the *wt*PCAT1 is proteolytically active. Biochemical analysis of the cryo-EM sample shows that a fraction of the substrate has been cleaved within the time window of sample preparation (*SI Appendix*, Fig. S4*C*). Consistently, the density inside the TM cavity is separated from both leader peptides, indicating that the cargo peptide inside the TM cavity has been freed from its leader peptide. In contrast to the recent electron paramagnetic resonance study (11), we do not observe any specific interactions between the cargo and residues lining the TM cavity, supporting the previous conclusion that the substrate is recruited by the PEP domain, and the TM cavity serves as a large conduit to accommodate the cargo (6).

The cryo-EM structures were determined in the presence of an ATP-regenerating system to retain the ATP concentration at 10 mM. Densities consistent with ATP and Mg^2+^ were observed in all three reconstructions (Fig. 3*D*). Different from the NBD-dimerized structure, in the NBD-separated conformations, ATP binds to the Walker A/B motifs of one NBD, distant from the ABC signature motif of the opposite NBD. It is likely that these structures represent the conformations en route to the ATP-bound, NBD-dimerized state.

## Discussion

**Fig. 4.**
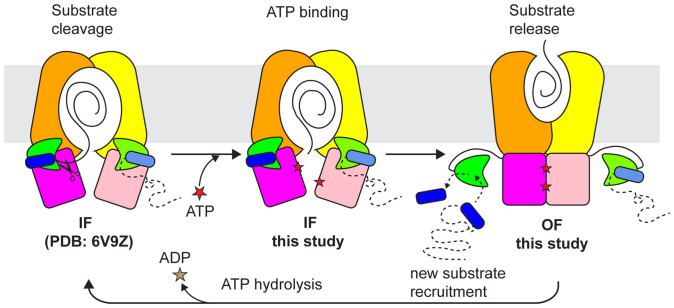
A model of the PCAT1 transport cycle. The substrate-bound IF state is based on the C21A mutant structure. The ATP-bound IF conformation is based on the structures determined under ATP turnover conditions. The NBD-dimerized OF state is based on the structure determined in equilibrium with ATP. We propose that dissociation of the PEP domains from the transporter core also enables new substrate recruitment in the OF conformation.

In this study, we determined the structures of *wt*PCAT1 in the presence of substrate and ATP with and without Mg^2+^ ion. In the absence of Mg^2+^, PCAT1 predominately exhibits an OF conformation, in which the PEP domains are detached from the translocation pathway, the NBDs are dimerized with two ATP molecules poised for hydrolysis, and the cargo substrate has been released. Inclusion of Mg^2+^ to enable ATP hydrolysis resulted in completely different structures. The majority of PCAT1 adopts an IF conformation in which the NBDs are separated, the substrate is enclosed inside the TM cavity, and the PEP domains remain associated with the transporter core. These results, together with previous biochemical and structural information (5, 6), support a mechanism to explain how substrate processing, translocation, and ATP hydrolysis are coupled in the PCAT1 transport cycle (Fig. 4). In the IF states, two substrates may be recruited at the same time, but only one is inserted into the TM cavity. The proteolytic cleavage takes place near the intracellular opening, freeing the cargo from the leader peptide, which remains attached to the PEP domain. ATP binding stabilizes an NBD-dimerized OF conformation in which the substrate is released to the extracellular space and the PEP domains are disengaged from the transporter core. Dissociation of the PEP domains diminishes proteolytic activity of PCAT1, thereby preventing cleavage of substrates before positioning them inside the translocation pathway. Finally, ATP hydrolysis leads to NBD dissociation, thus resetting the transporter to the IF state.

**Fig. 5.**
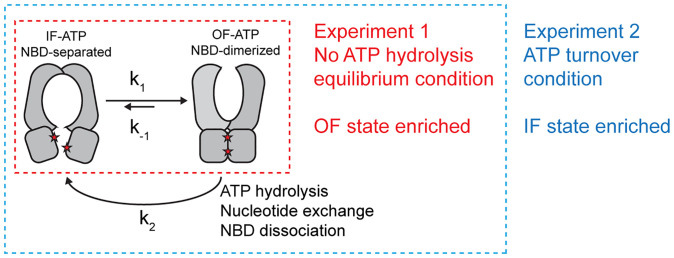
A simplified model to interpret the cryo-EM experiments.

The marked difference in the conformational distributions observed with and without Mg^2+^ reveals energetic and kinetic properties of PCAT1. To analyze the cryo-EM observations, we simplified the transport cycle with a two-state model by subsuming multiple NBD-separated IF conformations into a single state (Fig. 5). In addition, because PCAT1 catalyzes the hydrolysis of ATP with a Michaelis constant (*K_m_*) of 0.23 mM (5) and 10 mM ATP was included in the sample, we assume that all IF conformations contain ATP. In this model, the transition from NBD-separated to NBD-dimerized states involves an isomerization step (*k*_1_). The NBD-dimerized OF PCAT1 has two routes to return to the NBD-separated state: through isomerization (*k*_−1_) or through ATP hydrolysis followed by NBD dissociation and nucleotide exchange. A composite rate constant *k*_2_ is used to describe the latter route.

Assuming the cryo-EM samples were vitrified after the system reached steady state, then the probability of observing the NBD-separated IF state (*P_IF_*) versus that of the NBD-dimerized OF state (*P_OF_*) is determined by Eq. **1**:

[1] $$\frac{P_{IF}}{P_{OF}}=\frac{k_{2}+k_{-1}}{k_{1}}.$$

In the first experiment, Mg^2+^ is omitted to prevent ATP hydrolysis, that is, *k*_2_ = 0 (12). Under this condition, the IF and OF states are in equilibrium, and the Boltzmann distribution law states that the conformational distribution depends on the energy difference between the two states (E_OF_ − E_IF_):

[2] PIFPOF =k-1k1 = e(EOF-EIF)kBT,

where *k*_B_ is the Boltzmann constant, and *T* is the absolute temperature of the system.

Cryo-EM analysis only resolved the NBD-dimerized OF conformation, indicating that *P_OF_* ≫ *P_IF_*. Using Eq. **2**, we conclude that the energy of the OF conformation is lower than that of the IF conformation. The fact that *P_OF_* ≫ *P_IF_* also means *k*_1_ ≫ *k*_−1_; that is, in the presence of ATP, the NBD-dimerized OF conformation is stable, and the reverse isomerization (*k*_−1_) is much slower than that of NBD dimerization.

In the second experiment, addition of Mg^2+^ enables energy dissipation from ATP hydrolysis, shifting the system out of equilibrium. Under this condition, the Boltzmann distribution no longer applies. As we learned from the first experiment, where *k*_1_ ≫ *k*_−1_, Eq. **1** can be approximated as

[3] $$\frac{P_{IF}}{P_{OF}}=\frac{k_{2}+k_{-1}}{k_{1}}\;=\frac{k_{2}}{k_{1}}+\;\frac{k_{-1}}{k_{1}}\;≅\;\frac{k_{2}}{k_{1}}.$$

The 3D classification analysis in the presence of Mg^2+^ shows that ∼83% of the particles exhibit the NBD-separated IF conformations, and about 14% are in the NBD-dimerized OF conformations (*SI Appendix*, Fig. S2). If we were to make approximate inference from this result, then the ratio of *k*_2_/*k*_1_ is about 6. In other words, resetting the NBD-dimerized OF conformation to the NBD-dissociation IF conformation (*k*_2_) takes place six times faster than formation of the NBD-dimerized OF conformation (*k*_1_).

The above analyses made a few assumptions that are yet to be experimentally validated. First, we assume that the omission of Mg^2+^ completely abolished ATP hydrolysis in the time frame of cryo-EM sample preparation. Second, the addition of Mg^2+^ in the second experiment does not fundamentally change the result from the first experiment that *k*_1_ ≫ *k*_−1._ Finally, we assume that in both experiments, neither the IF nor the OF transporter was preferentially denatured on the cryo-EM grids.

Recent advances in cryo-EM have enabled us to study the conformational landscape of dynamic molecules such as ABC transporters. It is important to keep in mind that which conformation dominates under ATP turnover conditions is a kinetic property specified by the relative rates of transitions in the transport cycle. In contrast, in equilibrium conditions where ATP hydrolysis is abolished, the relative abundance of conformations is a thermodynamic property of the system determined by the relative energies of states. Our analyses of PCAT1 give rise to two conclusions: 1) in the presence of ATP, the NBD-dimerized conformation is the lowest energy state, and 2) in the PCAT1 transport cycle, the rate-limiting step is NBD dimerization.

So far, the structures of many ABC transporters determined under equilibrium conditions using nonhydrolyzable ATP analogs or catalytic-deficient mutants resulted in an NBD-dimerized conformation (13–21). Thus, it is likely a general property of ABC transporters that in the presence of ATP, the NBD-dimerized conformation is the lowest energy state.

In contrast, under ATP turnover conditions, different conformational distributions were observed for different ABC transporters. The multidrug transporter ABCG2 exhibits only IF, NBD-separated conformations (22), indicating that similar to PCAT1, NBD dimerization is rate limiting. The opposite result was obtained with MRP1, which adopts the NBD-dimerized, OF conformation in the presence of ATP and Mg^2+^ (23). However, the *Thermus thermophilus* multidrug-resistance proteins A and B (TmrAB) exhibit both NBD-separated and NBD-dimerized conformations (10). These different results suggest that the kinetic bottleneck of the transport cycle varies from transporter to transporter.

## Materials and Methods

### Protein Expression and Purification.

Wt and E648Q PCAT1 were expressed with an N-terminal glutathione-*S*-transferase (GST) tag and purified as previously described (6). Briefly, the constructs were expressed in *Escherichia coli* strain BL21(DE3) codon plus (RIL) cells. The cells were lysed in buffer containing 1% *n*-dodecyl-β-d-maltoside (Anatrace), 50 mM Tris pH 7.0, 500 mM NaCl, 10% glycerol, and 5 mM dithiothreitol (DTT). The supernatant was applied to the glutathione Sepharose 4B affinity resin followed by extensive washing with 50 mM Tris pH 7.0, 500 mM NaCl, 10% glycerol, 5 mM DTT, and 2 mM *n*-undecyl-β-d-maltopyranoside (UDM; Anatrace). The GST tag was removed by tobacco etch virus (TEV) protease, and the protein was further purified by gel filtration chromatography using a Superdex 200 increase column (GE Healthcare) in a buffer containing 50 mM Tris pH 7.0, 150 mM NaCl, and 2 mM UDM.

The substrate CtA was expressed and purified as previously described (6). Briefly, CtA-containing inclusion bodies were solubilized in 8 M urea, 50 mM Tris pH 7.0, 150 mM NaCl, and 10% glycerol. The denatured protein was purified on cobalt affinity resin (Clontech Laboratories) and refolded by dialysis. The His tag was removed by TEV protease, and the protein sample was further purified by gel filtration chromatography (Superdex 75 HiLoad 16/60, GE Healthcare).

### Cryo-EM Sample Preparation, Data Collection, and Processing.

Purified *wt*PCAT1 (43 μΜ) was mixed with twofold molar excess of CtA (86 μM) in the presence of an ATP-regenerating system (0.1 mg/mL creatine phosphokinase and 10 mM creatine phosphate). Upon addition of 10 mM ATP in the presence or absence of 10 mM Mg^2+^, the sample was immediately applied onto glow-discharged holey carbon grids (Quantifoil gold R1.2-1.3), incubated for 20 s at 100% humidity, and blotted with filter paper for 3 s before being plunge-frozen into liquid ethane using a Vitrobot Mark IV (FEI). Data were collected on the Titan Krios Transmission Electron Microscope (FEI) with a K2 Summit direct electron detector (Gatan).

The procedures for image processing are summarized in *SI Appendix*, Figs. S1 and S2 and Tables S1 and S2. Movie frames were corrected for gain reference and binned by two to yield a pixel size of 1.03 Å/pixel. Subframe alignment was carried out using MotionCorr2, and the contrast transfer function (CTF) was estimated using Gctf software (24). Particle picking, two-dimensional (2D) classification, and 3D classification were performed using RELION-3 (25). Each class was then subjected to one round of 3D refinement followed by masked 3D refinement. Two cycles of CTF refinement, Bayesian polishing, and masked 3D refinement in RELION-3 were then performed to improve the map quality (26). The final round of refinement was performed without symmetry imposed in CryoSPARC 2 software using nonuniform refinement (27).

Finally, a maximum likelihood density modification procedure was used to improve the side chain and nucleotide densities (28). Two independent half maps and a polyalanine model without nucleotide ligands were used as inputs for the density modification procedure implemented in Phenix. The output cryo-EM map was used for model building and figure preparation.

### Model Building and Refinement.

The structure of ATP-bound PCAT1 was built based on the crystal structure of apo-PCAT1 (Protein Data Bank [PDB]: 4RY2). Polyalanine models were placed where side chain densities were invisible. The final model consists of PCAT1 residues 9 to 722 and two molecules of ATP.

The structures of the IF conformations under active turnover conditions were built by docking the cryo-EM structure of CtA–PCAT1 complex (PDB: 6V9Z) into the cryo-EM maps using rigid body fitting in Chimera, followed by manual adjustments in Coot. The final models consist of PCAT1 residues 9 to 722, CtA residues 14 to 24, two ATP molecules, and two Mg^2+^ ions.

All models were refined against their final half maps using the real-space refinement procedure in Phenix followed by iterative cycles of reciprocal-space refinement in Refmac and manual rebuilding in COOT (29, 30). MolProbity was used to assess the quality of the final model (31). Structural model validation was performed using SPIDER (32).

### Figure Preparation.

Figures were prepared with PyMOL (33), UCSF Chimera (34), and UCSF ChimeraX (35).

## Supplementary Material

Supplementary File

## Data Availability

Atomic coordinates have been deposited in PDB (accession nos. 7T54 [OF conformation] and 7T55, 7T56, and 7T57 [IF conformations]). Cryo-EM density maps have been deposited in the Electron Microscopy Data Bank (accession nos. EMD-25694 [OF conformation] and EMD-25695, EMD-25696, and EMD-25697 [IF conformations]).
