# Supplementary material for: Structures of the peptidase-containing ABC transporter PCAT1 under equilibrium and nonequilibrium conditions
Source: Proc Natl Acad Sci U S A. 2022 Jan 24;119(4):e2120534119. doi: 10.1073/pnas.2120534119 (PMC8794836; doi:10.1073/pnas.2120534119)
Supplement: Supplementary File [file pnas.2120534119.sapp.pdf]

Supplementary Materials for

Structures of the peptidase-containing ABC transporter PCAT1 under equilibrium and non-equilibrium conditions

Virapat Kieuvongngam[1] and Jue Chen[1,2,*]

[1]Laboratory of Membrane Biology and Biophysics, The Rockefeller University, New York, NY 10065, USA.

[2]Howard Hughes Medical Institute, Chevy Chase, MD 20815, USA.

*Correspondence should be addressed to (juechen@rockefeller.edu).

This PDF file includes:

Figs. S1 to S4

Table S1 and S2

References

Supporting information

Supplemental Figures

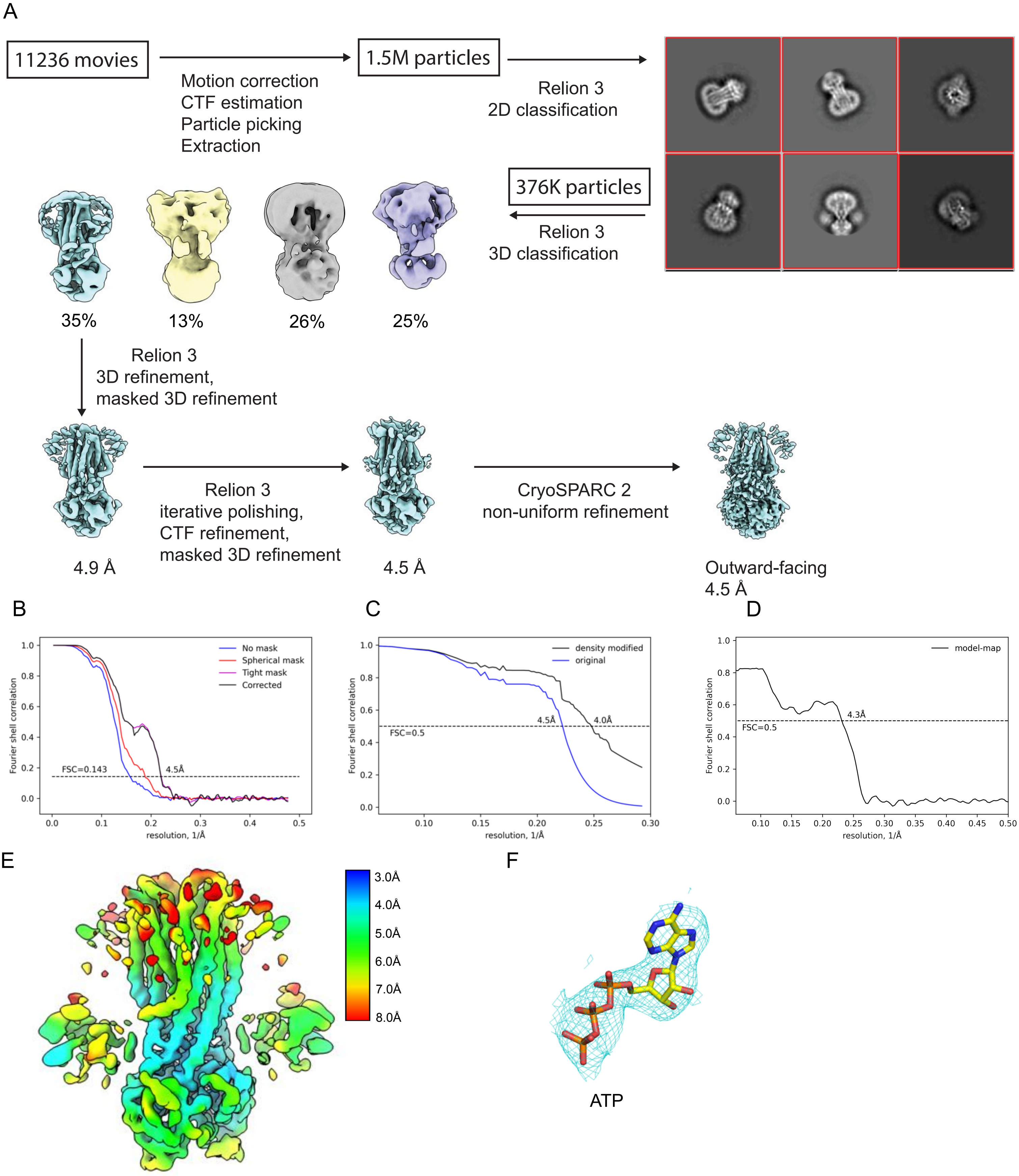

Fig. S1. Cryo-EM reconstruction of the Mg2+-free dataset.
(A) Flowchart of the cryo-EM data processing workflow. **(B)** Fourier shell correlation (FSC) curves for the two independent half maps used in the final reconstruction, calculated in CryoSPARC2 with or without masks. The "Corrected" FSC curve is calculated using a tight mask with correction for overfitting by noise substitution procedure (1). **(C)** Estimated resolution dependent map quality (FSCref) of the original map and the density modified map performed in PHENIX (2). **(D)** Model validation showing model-to-map fit of the density modified full map and the model. **(E)** Local resolution estimation by CryoSPARC2. **(F)** Cryo-EM density of the ATP molecule.

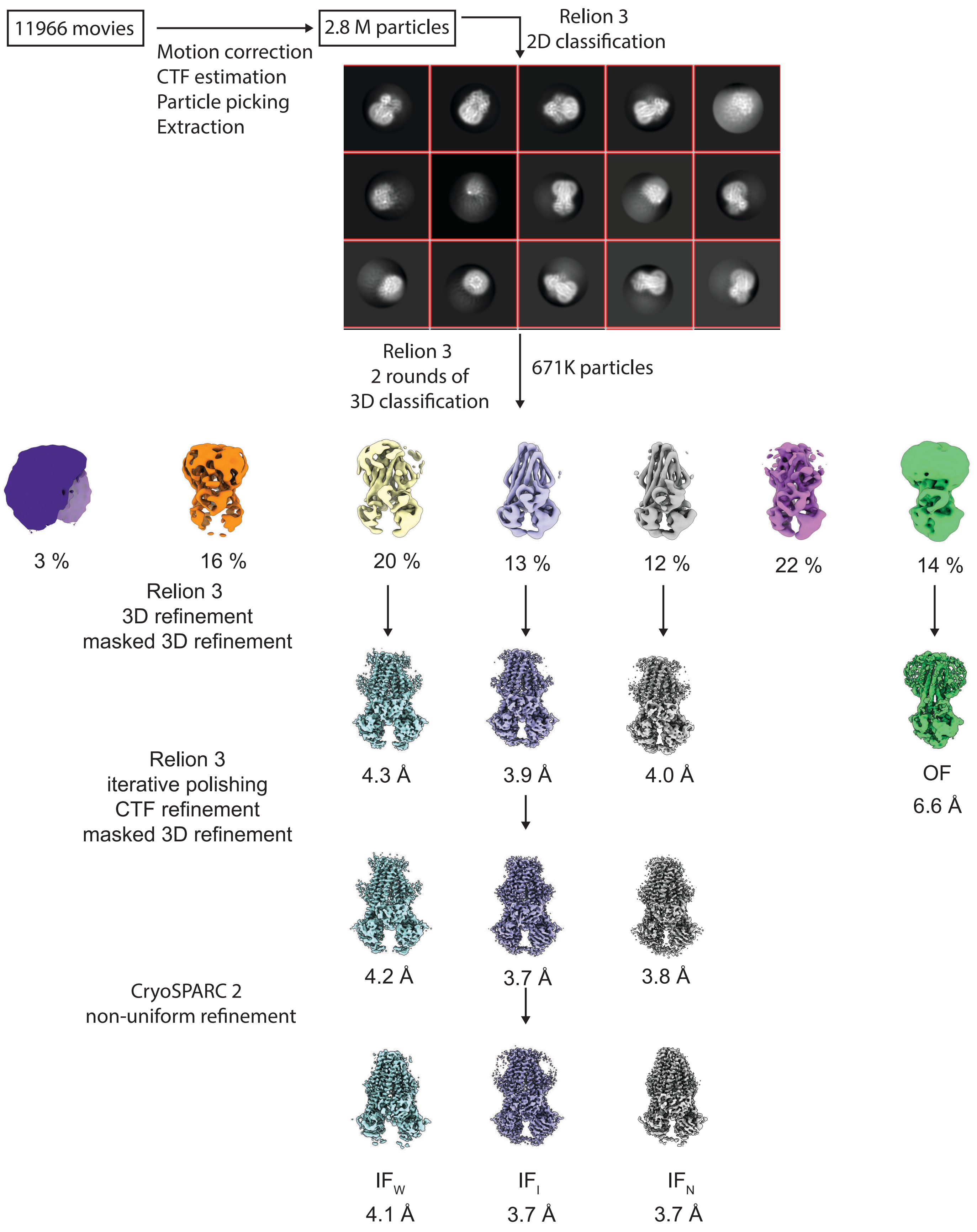

Fig. S2. Cryo-EM image processing workflow of the ATP turnover dataset.

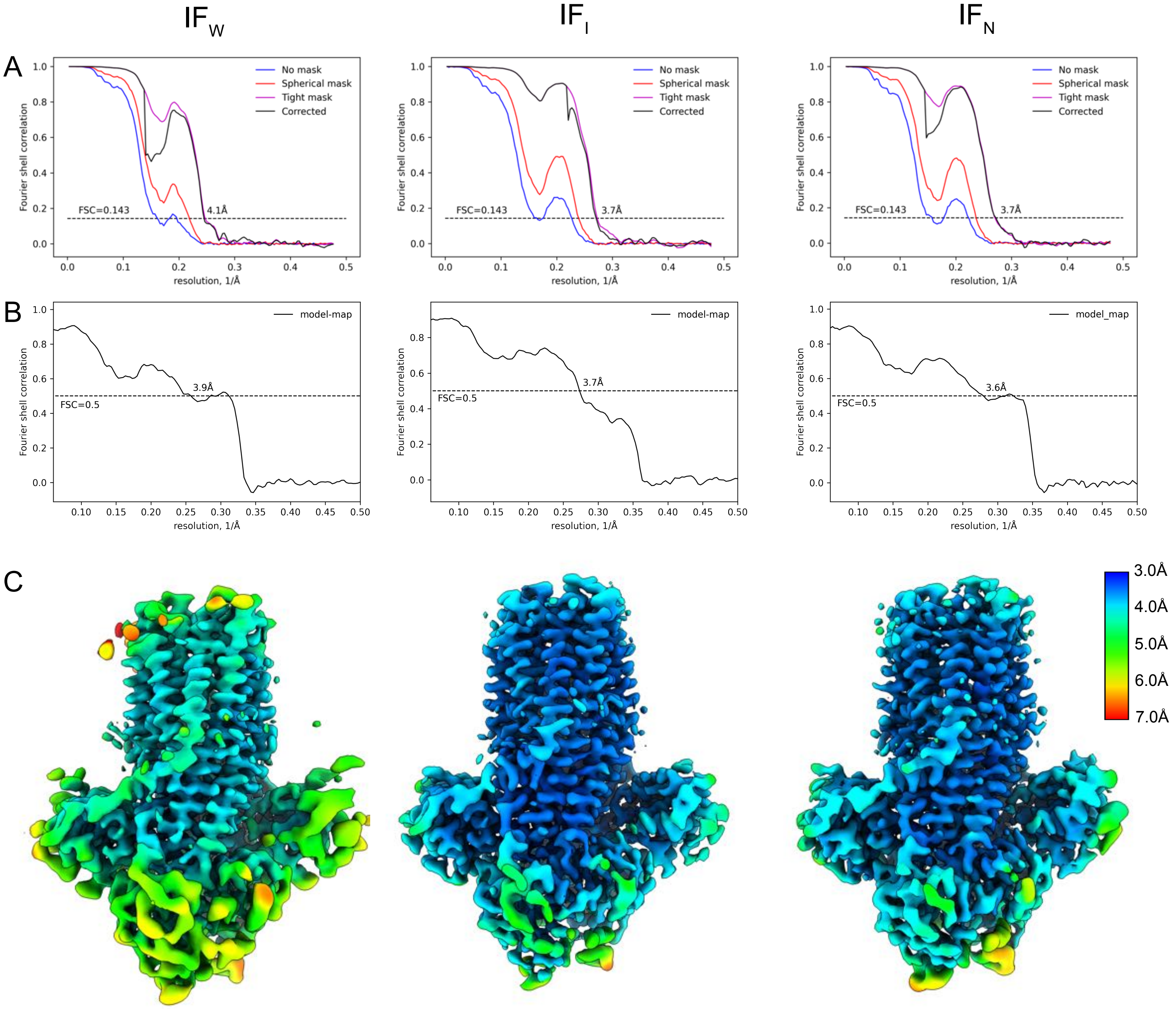

Fig. S3. Quality of the cryo-EM reconstruction of the three IF structures.
(A) FSC curves for the two independent half maps, calculated in CryoSPARC2.
(B) Model validation showing model-to-map fit of the full map and the model.
(C) Local resolution estimation of the IF conformations by CryoSPARC2.

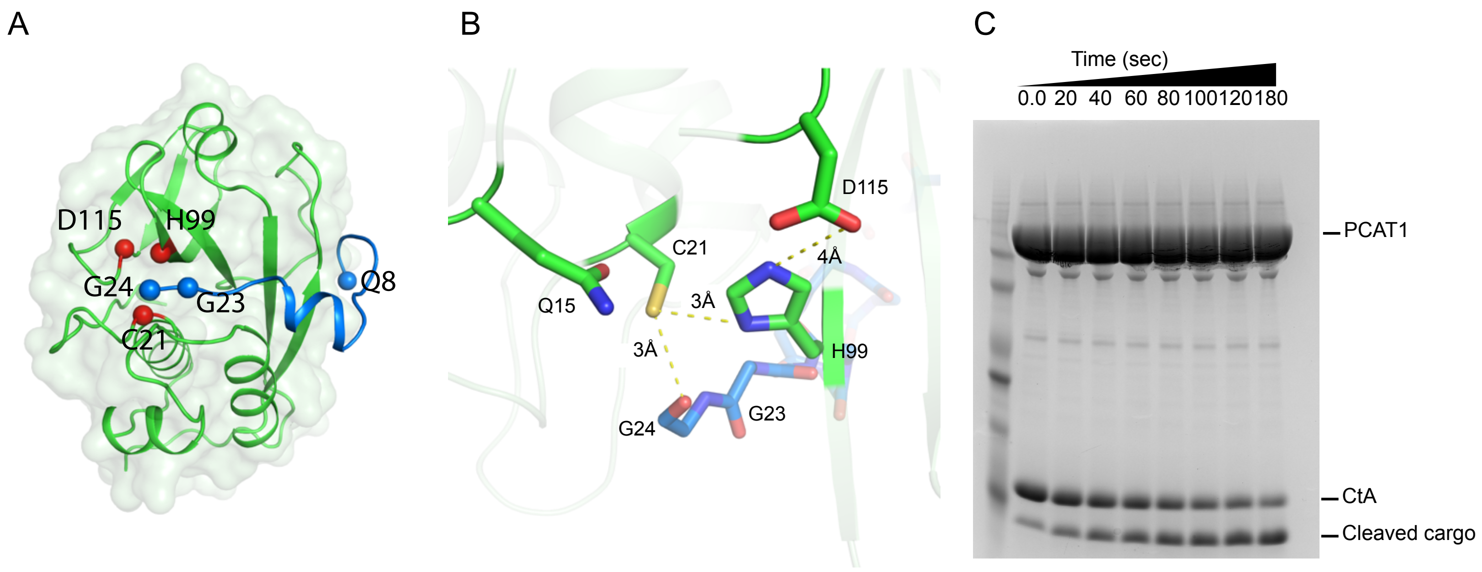

Fig. S4. Proteolytic activity of PCAT1 under ATP turnover condition.
(A) The structure of the PEP domain (green) with a bound leader peptide (blue).
The catalytic triad residues (red spheres) and the CtA double glycine motif (blue spheres) are indicated.
(B) A zoomed-in view of the catalytic site. The side chains of the catalytic residues are shown in green.
The backbone of the leader peptide is shown in blue.
(C) Substrate cleavage assay recapitulating the cryo-EM sample preparation condition
showing that the substrate is being processed within the time window of grid preparation.

Table S1. Summary of Cryo-EM data and structure refinement statistics in Mg^{2+} free condition

**Data collection**	
Microscope	Titan Krios (FEI)
Voltage (kV)	300
Detector	K2 (Gatan)
Pixel size (Å)	1.03
Defocus range (μM)	0.8-2.2
Movies	5994
Frames/movie	50
Dose rate (electron/pixel/s)	8
Total dose (electrons/$Å^2$)	75
**Refinement**	
Number of particles	98523
Resolution (Å)	4.5
**Model composition**	
Non-hydrogen atoms	8562
Protein residues	1458
ATP	2
Mg^{2+}	0
**Validation**	
**RMS deviation**	
Bond lengths (Å)	0.014
Bond angles (∘)	1.70
**Ramachandran**	
Favored (%)	95.53
Allowed (%)	4.40
Disallowed (%)	0.07
**Rotamer**	
Favored (%)	98.40
Allowed (%)	0.92
Disallowed (%)	0.68

Table S2. Summary of cryo-EM data and structure refinement statistics under active turnover condition

**Data collection**			
Microscope		Titan Krios (FEI)	
Voltage (kV)		300	
Detector		K2 (Gatan)	
Pixel size (Å)		1.03	
Defocus range (μM)		0.8-2.2	
Movies		11966	
Frames/movie		50	
Dose rate (electron/pixel/s)		8	
Total dose (electrons/Å^2)		75	
**Refinement**			
**Conformation**	**IF_W**	**IF_I**	**IF_N**
Number of particles	134772	88549	80156
Resolution (Å)	4.1	3.7	3.7
**Model composition**			
Non-hydrogen atoms	11460	11460	11444
Protein residues	1458	1458	1458
ATP	2	2	2
Mg^{2+}	2	2	2
**Validation**			
**RMS deviation**			
Bond lengths (Å)	0.0028	0.0025	0.0027
Bond angles (∘)	1.29	1.22	1.26
**Ramachandran**			
Favored (%)	97.05	96.57	98.64
Allowed (%)	2.88	3.29	0.64
Disallowed (%)	0.07	0.14	0.72
**Rotamer**			
Favored (%)	98.88	98.64	97.12
Allowed (%)	0.88	0.64	2.74
Disallowed (%)	0.24	0.72	0.14

Reference:

1. S. Chen, *et al.*, High-resolution noise substitution to measure overfitting and validate resolution in 3D structure determination by single particle electron cryomicroscopy. *Ultramicroscopy* **135**, 24–35 (2013).
2. T. C. Terwilliger, S. J. Ludtke, R. J. Read, P. D. Adams, P. v Afonine, Improvement of cryo-EM maps by density modification. *Nature Methods* **17**, 923–927 (2020).
